# The Triangle of Nonalcoholic Fatty Liver Disease, Metabolic Dysfunction, and Periodontitis: Analysis of the Dental, Oral, Medical and Epidemiological (DOME) Records-Based Nationwide Research

**DOI:** 10.3390/metabo12121212

**Published:** 2022-12-02

**Authors:** Doron Ram, Asaf Wilensky, Dorit Zur, Galit Almoznino

**Affiliations:** 1 In Partial Fulfillment DMD Thesis, Faculty of Dental Medicine, Hebrew University of Jerusalem, Jerusalem 91120, Israel; 2 Faculty of Dental Medicine, Hebrew University of Jerusalem, Israel; Department of Periodontology, Hadassah Medical Center, Jerusalem 91120, Israel; 3 Medical Information Department, General Surgeon Headquarter, Medical Corps, Israel Defense Forces, Tel Hashomer 02149, Israel; 4 Faculty of Dental Medicine, Hebrew University of Jerusalem, Israel; Big Biomedical Data Research Laboratory; Dean’s Office, Hadassah Medical Center, Jerusalem 91120, Israel; 5 Faculty of Dental Medicine, Hebrew University of Jerusalem, Israel; Department of Endodontics, Hadassah Medical Center, Jerusalem 91120, Israel; 6 Faculty of Dental Medicine, Hebrew University of Jerusalem, Israel; Department of Oral Medicine, Sedation & Maxillofacial Imaging, Hadassah Medical Center, Jerusalem 91120, Israel

**Keywords:** fatty liver, nonalcoholic fatty liver disease (NAFLD), metabolic syndrome, metabolic dysfunction-associated fatty liver disease (MAFLD), diabetes type 2, hyperlipidemia, hypertension, obesity, periodontitis, dental caries, decayed teeth, electronic medical record, electronic dental record

## Abstract

This study aimed to analyze the associations of nonalcoholic fatty liver disease (NAFLD) with dental parameters, while controlling for socio-demographics, health-related habits, and each of the metabolic syndrome (MetS) components, consequences, and related conditions among a nationally representative sample of young and middle-aged adults. To that end, we analyzed data from the dental, oral, medical epidemiological (DOME) cross-sectional records-based study that combined comprehensive socio-demographic, medical, and dental databases of a nationally representative sample of military personnel. Included were 132,529 subjects aged 18–50 who attended military dental clinics for one year. The prevalence of NAFLD in the study population was 0.7% (938/132,529). The following parameters maintained a statistically positive association with NAFLD in the multivariate analysis (from highest to lowest OR): male sex (OR = 3.91 (2.29–6.66)), hyperlipidemia (OR = 3.69 (2.75–4.95)), diabetes Type 2 (OR = 3.14 (2.21–4.46)), hypertension (OR = 1.67 (1.30–2.14)), periodontitis (OR = 1.42 (1.06–1.89)), body mass index (BMI) (OR = 1.15 (1.13–1.18)), and age (OR = 1.08 (1.06–1.09)). The multivariate analysis established a profile of the “patient vulnerable to NAFLD”, including older age, male sex, and other MetS components, including diabetes type 2, hypertension, hyperlipidemia, BMI, and periodontitis. This profile aligns with the current new definition of metabolic dysfunction-associated fatty liver disease (MAFLD). We also analyzed the associations of the sum of the standard dental unit (SDU) scores of planned (SDU-P) and delivered (SDU-D) dental procedures per patient with NAFLD using receiver operating characteristic (ROC) analysis. The SDU-P (planned) score exhibited excellent discrimination for NAFLD (area under the curve (AUC) = 0.718 (0.703–0.734)). Overall, the results confirmed the hypothesis of this research, i.e., that NAFLD is associated with dental morbidity, particularly with periodontitis.

## 1. Introduction

Non-alcoholic fatty liver disease (NAFLD) is the most common chronic liver disease worldwide, representing the hepatic manifestation of metabolic syndrome (MetS) [1]. Given this current understanding, in 2020, the Asian Pacific Association for the Study of the Liver (APASL), including a panel of international experts from 22 countries, redefined NAFLD as metabolic dysfunction-associated fatty liver disease (MAFLD) [2]. NAFLD affects about a quarter of the global adult population, ranging from 5–42%, depending on the diagnostic criteria [1,2,3], and poses a major health and economic burden to all societies, yet it has no approved pharmacotherapy [1,2]. The criteria for MAFLD are based on evidence of hepatic steatosis, in addition to one of the following three criteria: overweight/obesity, presence of type 2 diabetes mellitus, or evidence of metabolic dysregulation [2]. While NAFLD is diagnosed per exclusion, MAFLD applies positive diagnostic criteria and does not exclude alcohol intake or other liver diseases [2]. However, given its complex pathophysiology, the APASL states that it is unlikely that a single diagnostic test will become available, as was the case for MetS, which has multiple definitions [4].

NAFLD also shares some risk factors with dental conditions, including periodontitis and dental caries. For example, the consumption of simple sugars (glucose and fructose) is a factor leading to NAFLD and dental caries, and several hypotheses link NAFLD and periodontitis, through periodontal pathogens, inflammatory mediators, and oxidative stress [5]. Periodontal disease is characterized by the inflammatory destruction of the tooth-supporting tissues, including the cement, periodontal ligament, and alveolar bone. Periodontitis is the sixth most common human disease, with a global prevalence of almost 50% of the adult population. The severe form affects 9.8% (796 million people) globally [6,7]. Periodontitis is the leading cause of tooth loss in the adult population worldwide, with major negative consequences on masticatory function, thereby affecting their nutrition, esthetics, and overall quality of life [8]. Dental caries is also a prevalent disease, with untreated caries of the permanent dentition considered the most prevalent condition worldwide, with a global prevalence of 35% for all ages combined [9]. Dental caries is a diet-dependent, transmissible microbiologically mediated disease that also follows an infectious and chronic disease model [10]. Due to its predominance, dental caries is considered the most important oral disease and is of medical, social, and economic importance [11].

Associations between dental problems and MetS have been previously investigated, including by our group [12,13,14,15,16,17]. Over the last few decades, more than 60 different systemic conditions have been investigated in relation to periodontitis [18]. A significant body of evidence supports independent associations between severe periodontitis, including diabetes, cardiovascular disease, chronic obstructive pulmonary disease, and chronic kidney disease, and even with all-cause and cardiovascular mortality in several populations [19]. In particular, a “bidirectional” relationship has been ascertained for the associations between periodontitis and diabetes, leading to the designation of periodontitis as the “sixth complication of diabetes mellitus”[20]. The associations between dental status and NAFLD have been less studied, compared to the associations with diabetes and cardiovascular diseases. The available literature demonstrates conflicting results, with some reporting that NAFLD is positively associated with periodontitis and tooth loss [5,21,22,23,24], while other studies failed to replicate the previously reported associations between periodontitis and NAFLD [25,26].

These conflicting results could be attributed to the limitations of the published studies, such as heterogeneity in the definitions of dental and systemic diseases and the presence of possible confounders that were not always considered. For example, there are well-known common risk factors for multiple chronic diseases, including increased age, socioeconomic status, smoking, sugar consumption, and obesity [27]. Considering these limitations, there is a need for large-scale epidemiological studies on the association between NAFLD and dental morbidities that employ a rigorous protocol regarding dental and medical disease definitions and consider the existence of many possible confounders. It is also important to study the associations between NAFLD and dental morbidities in the context of the MetS cluster, and periodontitis should be studied in the context of other dental and oral conditions that might exist concomitantly in an individual, i.e., within the context of “dental cluster”, as we previously described [15]. Moreover, there is a need to study the association between NAFLD and the dental burden, which is a function of the number of both the planned and the delivered dental procedures, which, to the best of our knowledge, has not been studied yet. Particularly, it is important to assess the associations between dental status and NAFLD among young and middle-aged adults, a less studied population in this context.

To address the gap in the literature, the primary objective of this study was to test the hypothesis that poor dental status, as reflected by the presence of periodontitis and by a higher prevalence of dental treatment needs, will be the predictor of the research outcome of NAFLD diagnosis. Specific objectives were to:

A. Measure the prevalence of NAFLD and study the associations of NAFLD, with the prevalence of the following planned and delivered dental procedures: (1) fillings, (2) endodontic treatments, (3) post-treatment, (4) crowns, (5) extractions, (6) periodontal disease, and (7) missing teeth, as well as with the medical and dental attendance patterns. This specific goal will enable us to address the “dental cluster”.
B. To address possible confounders, we aim to further explore the associations of NAFLD with dental status in a multivariate model controlling for (1) socio-demographic parameters, (2) health-related risk habits, and (3) each of the MetS components and consequences and related conditions, including diabetes type 2, hypertension, hyperlipidemia, impaired glucose tolerance (IGT), obesity, cardiovascular disease, S/P stroke, S/P transient ischemic attack (TIA), obstructive sleep apnea (OSA), and (5) auxiliary test results, including the blood tests used in the assessment of MetS related conditions. This specific goal will enable us to address the “MetS cluster”.

## 2. Methods

### 2.1. Data Source

The current study used data from the “The Dental, Oral, Medical Epidemiological (DOME)” study [14,15,17,28,29,30,31]. The DOME study is based on a structured records-based repository that combines comprehensive socio-demographic, medical, and dental databases of a nationally representative sample of military personnel from the Israel Defense Forces (IDF) [30]. The protocol and methods of data collection of the DOME study were previously detailed in-depth [30]. 

**Ethical approval.** Approval for the study was obtained from the Medical Corps Institutional Review Board (IRB number: IDF-1281-2013). Considering the retrospective study design, including anonymous records analyses, an exemption from written informed consent was given by the IRB.

**Inclusion criteria:** The socio-demographic, medical, and dental records of all IDF military personnel, aged 18 years and older of both sexes, who visited military dental clinics of the IDF between 1 January 2015 and 1 January 2016, for which there are records in the socio-demographic medical dental patient record (DPR).

**Exclusion criteria:** Subjects with a lack of data in these databases.

The research consisted of 132,529 records of patients who fulfilled the eligibility criteria.

As explained in previous publications, a remarkable opportunity exists in Israel for research on dental-systemic associations by utilization of the comprehensive information restored in the military databases and captured by the DOME repository. The Israeli military population is large and constitutes a credible data source for epidemiological studies among young and middle-aged adults [14,15,17,28,29,30,31,32]. This is partially due to conscription in Israel for all Jewish, Druze, or Circassian citizens aged 18 and older [30]. Of importance is the fact that the service in the IDF includes individuals with a complex medical background, excluding subjects who are unfit for service, due to severe health reasons (physical or mental), and even for military exemption recipients, there is an option to apply for volunteering [33,34]. Dental care services are included in the comprehensive medical care basket and are provided to IDF military personnel free of charge [30,35]. In the context of this study, in general, the military population does not include heavy consumers of alcohol, since it is forbidden.

### 2.2. Data Collection

A full description of the data collection of the DOME study had been published previously [30]. Briefly, the DOME structured repository captures 3 military electronic databases: *Dental Patient Record* (DPR), medical (i.e., computerized patient record (CPR)), and socio-demographic computerized systems that store personal socio-demographic profiles and dental and medical records of all military personnel [30].

### 2.3. Study Variables

We analyzed the associations of NAFLD as dependent, with various independent, variables. Definitions of the variables available in the DOME repository have been detailed in the DOME protocol and methods paper previously [30], and will be described briefly.

**Definitions of Socio-Demographic Parameters.***Age (years), sex (men/women), duration of service (months), education (high school and below/technical college/academic), locality of residence (urban Jewish, urban non-Jewish, rural), socio-economic status (SES), as retrieved from the Israeli Ministry of the* Interior records (low (1st–4th deciles)/medium (5th–7th deciles)/high (8th–10th deciles)), and birth country (western Europe, eastern Europe, Asia, Ethiopia, Africa, North America, South America, Israel) [30].

**Definitions of Health-Related Habits.** Based on self-reporting: current smoker (yes/no), teeth brushing at least once a day (yes/no), consumption of cariogenic diet (snacks and/or sweets consumption between or instead of meals (yes/no)), consumption of sweetened beverages (sweet drinks consumption above one cup a day (yes/no)) [30].

**Definitions of Medical and Dental Attendance Patterns.**

Measurement health care utilization during the study period included: appointments with general physicians, periodontal specialists, and oral medicine specialists, the total number of dental appointments, scaling and root planning appointments, and non-attendance to scheduled dental appointments [30].

**Definitions of Medical Diagnoses and Auxiliary Test Results.** The medical diagnoses of MetS components, consequences, and associated morbidities as well as the auxiliary test results were retrieved from the CPR as described previously [14,15,30] and included: 

**Medical Diagnoses:** Diagnoses on the CPR are based on the ICD-9-CM:

1. The dependent variable—nonalcoholic fatty liver disease (NAFLD), equivalent to 2015 ICD-9-CM Diagnosis Code 571.8: other chronic nonalcoholic liver disease.
2. Other systemic conditions related to MetS were included as independent variables and were also based on ICD-9-CM diagnoses, including diabetes mellitus, impaired glucose tolerance (IGT), hyperlipidemia, hypertension, cardiovascular disease, obesity (BMI > 30 kg/m^2^), obstructive sleep apnea (OSA), S/P (status post) transient ischemic attack (TIA), and S/P stroke [14,15,30].

**Auxiliary Test Results:** The auxiliary test results retrieved from the CPR included blood tests used in the assessment of MetS components, as described previously weight (in kilograms), body mass index-BMI (weight/height^2^ (kg/m^2^)), C reactive protein-CRP (mg/L), glycated hemoglobin-HbA1c (%), fasting glucose (mg/dL), cholesterol (mg/dL), high-density lipoprotein-HDL (mg/dL), low-density lipoprotein-LDL (mg/dL), triglycerides (mg/dL), very low-density lipoprotein-VLDL (mg/dL), and non-HDL cholesterol (mg/dL) [14,15,30].

**Definitions of Dental Parameters.** Details on the standardized uniform codes employed in the DPR for each dental procedure and diagnosis can be found in previous publications [15,17,30]. Briefly, dental codes in the DPR are equivalent to the nomenclature used by the American Dental Association’s (ADA) current dental terminology (CDT) [36]. The dental procedures were drawn from the DPR as planned (i.e., treatment plan) and delivered (i.e., actual treatments that were performed) [15,17,30]. In addition to procedures, the DPR data include records of the presence of periodontal disease and a count of missing teeth for any reason (excluding wisdom teeth) [30]. The definition of periodontitis was detailed in the DOME protocol paper and was based on the American Academy of Periodontology from 2010 to 2018 (current data was collected in 2016). Furthermore, even before 2018, routinely collected data included age, smoking habits, and metabolic morbidity, including diabetes, and for those with diabetes, the glycated hemoglobin (HbA1c) was also measured. Moreover, due to possible pseudo pockets, radiographic bone loss was considered mandatory to establish a diagnosis of periodontitis radiographic bone loss, which is defined as the distance between the crestal margin and the cementoenamel junction, which is greater than 2 mm in more than one tooth, with no visible cause, such as faulty restoration, overhang, etc [30].

**Standard Dental Unit (SDU).** To employ the concept of “clustering” in dentistry, a standardized scoring system that quantifies dental needs and actual performance and their burden is needed. As we detailed previously, for several decades, the IDF dental corps routinely employs a standardized scoring system for dental procedures, termed the standard dental unit (SDU) [15]. The SDU score of each dental procedure represents the time and complexity of the executed procedure. For example, the SDU score of the procedure of “dental filling” (amalgam or composite) equals 1 SDU, “endodontic treatment of one root canal” equals 1.25 SDU, while the “crown” procedure equals 4 SDU. From the DOME repository, we computed for each patient the sum of planned dental procedures (SDU-P), as well as the sum of the actual treatments that were performed de facto (SDU-D). The definitions and comparisons of the SDU scores and CDT-related coding can be found elsewhere [15]. In this study, we also analyzed the associations of NAFLD with the SDU-P and SDU-D using ROC analysis (see below—statistical methods).

### 2.4. Statistical Methods

Statistical analyses were performed using SPSS software version 27.0 (IBM, Chicago, IL, USA). Descriptive statistics included the presentation of continuous variables as means and standard deviations (SD) and categorical variables as frequencies and percentages. 

**Univariate analysis.** Performed between NAFLD as a dependent variable and the independent variables using Pearson’s chi-square test or likelihood ratio test (for categorical parameters) and with a non-paired *t*-test for independent samples (for continuous variables). Odds ratios (OR) were calculated with linear regression for continuous variables, with binary logistic regression for categorical variables.

**Multicollinearity analyses.** Following the univariate analyses, we performed multicollinearity tests using linear regression to examine the collinearity of the independent variables. In a case where two or more variables were highly collinear, only one of them was included in the model, and it was decided by the context which of the variables will be included in the analysis. The variance inflation factors (VIFs), which are 1/tolerance, are calculated using linear regression analysis. While VIF < 10 is usually considered indicative of collinearity, in weaker models, VIF > 2.5 may be a cause for concern; therefore, the current study used VIF < 2.5 as a cutoff. 

**Multivariate analysis.** Following univariate analysis and collinearity statistics, a multivariate binary logistic regression analysis was performed for NAFLD as the dependent variable with statistically significant independent variables in the univariate analysis, which were not highly collinear.

**Receiver operating characteristic (ROC) analysis:** ROC analyses were performed on the SDU-P and SDU-D as predictors of NAFLD, and the area under the curve (AUC) was calculated to assess their discriminative ability. Cut-offs for AUC discrimination were as follows: (a) AUC ≤ 0.5: no discrimination, i.e., randomly; (b) 0.6 ≥ AUC > 0.5: poor discrimination, (c) 0.7 ≥ AUC > 0.6: acceptable discrimination, (d) 0.8 ≥ AUC > 0.7: excellent discrimination, and (e) AUC > 0.9: outstanding discrimination [37].

## 3. Results

### 3.1. The Associations between NAFLD and Socio-Demographic Parameters

The research consisted of 132,529 records of patients who fulfilled the eligibility criteria. There were 938 patients with NAFLD diagnosis; therefore, the prevalence of NAFLD was 0.7% (938/132,529). Table 1 presents a comparison of the socio-demographic characteristics between patients diagnosed with NAFLD and those without NAFLD. Compared to those without NAFLD, those with NAFLD had statistically significant positive associations, with the following parameters:

(1) Male sex (OR (95% CI) men vs. women: 6.29 (4.69–8.47)).
(2) Education: technical vs. high school education (OR = 26.73 (22.44–31.83)) and academic vs. high school education (OR = 17.17 (14.48–20.36)).
(3) SES: low vs. high SES (OR= 1.19 (0.86–1.65)) and medium vs. high SES (OR = 1.25 (1.09–1.43)).
(4) Birth country: being an immigrant from the following birth countries, compared to native Israelis: Africa (OR = 4.77 (2.60–8.73)), Asia (3.79 (2.18–6.61)), western Europe (OR = 1.49 (1.22–1.83)), eastern Europe (OR = 1.19 (0.70–2.02)).
(5) Older age (OR = 1.19 (1.18–1.20)).
(6) More time in service (OR = 1.14 (1.13–1.15)).

A statistically significant negative association between NAFLD and socio-demographic parameters was seen for:

(1) The locality of residence: urban Jewish vs. rural locality (OR = 0.26 (0.16–0.43)) and urban non-Jewish vs. rural locality (OR = 0.16 (0.09–0.28)).
(2) Birth country: being an immigrant from the following birth countries compared to native Israelis: Ethiopia (OR = 0.33 (0.13–0.80)), North America (OR = 0.25 (0.10–0.61)), and South America (OR = 0.76 (0.31–1.83)).

### 3.2. The Associations between NAFLD and MetS Components, Consequences, and Related Conditions

Table 2 presents a comparison of MetS-related conditions between patients diagnosed with NAFLD and those without NAFLD diagnosis. Those with NAFLD had a statistically significant positive association with the following MetS-related conditions (from the highest to lowest OR): obesity (OR = 45.45 (38.46–52.63)), hyperlipidemia (OR = 38.46 (33.33–45.45)), IGT (OR = 34.95 (22.47–54.37)), Diabetes Type 2 (OR = 33.33 (27.02–41.66)), obstructive sleep apnea (OR = 16.13 (11.76–22.22)), stroke (OR = 15.63 (8.62–27.78)), hypertension (OR = 13.51 (11.76–15.62)), transient ischemic attack (OR = 11.49 (5.92–22.22)), and cardiovascular disease (OR = 7.81 (6.62–9.17)).

### 3.3. The Associations of NAFLD with Auxiliary Test Results

Table 3 presents the associations of NAFLD with auxiliary test results, including blood tests used in the assessment of MetS-related conditions. Compared to those without NAFLD, those with NAFLD exhibited weak positive associations with all auxiliary test results, apart from HDL, which had a negative association with NAFLD. OR were close to 1, except for BMI (OR = 1.231 (1.217–1.245)) and HbA1c (OR = 1.277 (1.143–1.427)) (Table 3).

### 3.4. The Associations of NAFLD with Health-Related Habits and Medical and Dental Attendance Patterns

Table 4 presents a comparison of the health-related habits and medical and dental attendance patterns between patients with NAFLD and those without NAFLD.

**NAFLD and Health-Related Habits.** Compared to those without NAFLD, those with NAFLD had a statistically significant positive association with smoking (OR = 11.55 (10.10–13.2)). NAFLD diagnosis had a statistically significant negative association with teeth brushing once a day or more (OR = 0.50 (0.42–0.59)), consumption of a cariogenic diet (OR = 0.52 (0.43–0.62)), and the consumption of sweetened beverages (OR = 0.49 (0.41–0.59)) (Table 4).

**NAFLD and Attendance patterns to medical and dental services.** Compared to those without NAFLD diagnosis, those with NAFLD had statistically significant more: appointments with a general physician (OR = 1.016 (1.016–1.024)), scaling (OR = 1.41 (1.35–1.47)), root planning (OR = 1.35 (1.30–1.40)), appointments with oral medicine specialists (OR = 1.59 (1.38–1.82)) and with periodontal specialists (OR = 1.84 (1.70–1.99)), more total dental appointments (OR 1.030 (1.027–1.033)), and were more likely to not attend to scheduled dental appointments (OR = 1.068 (1.056–1.081)) (Table 4).

### 3.5. The Associations of NAFLD with Dental and Oral Status

Table 5 presents the associations between NAFLD and dental and oral status represented by diagnoses, as well as by dental procedures requirements and actual performance among the study population. Compared to those without NAFLD diagnosis, those with NAFLD had a statistically significant positive association with periodontitis diagnosis (OR = 2.41 (1.97–2.96)), presence of an oral soft tissue lesion in an oral examination (OR = 7.25 (5.81–9.09)), and all dental procedures requirements and actual performance, except for the number of teeth that required one surface amalgam filling (OR = 0.82 (0.76–0.88)), the number of teeth where one surface amalgam filling was performed (OR = 0.94 (0.87–1.03)), the number of teeth that required two amalgam fillings on two surfaces (0.79 (0.71–0.88)), and the total number of teeth that required fillings (OR = 0.97 (0.94–1.00)) (Table 5).

### 3.6. Receiver Operating Characteristic (ROC) Analyses of Planned and Delivered Standard Dental Units (SDU-P and SDU-D) as Predictors of NAFLD

**Figure 1:**
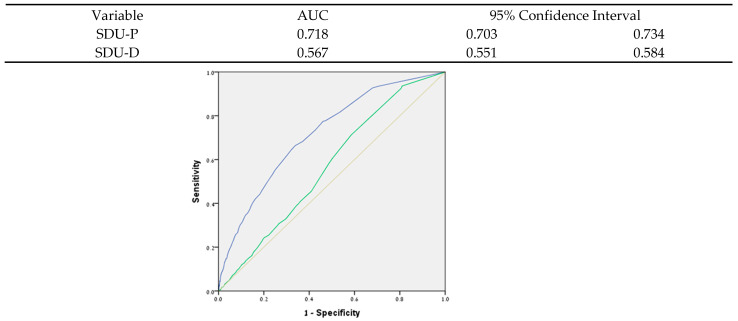
Receiver operating characteristic (ROC) analyses of planned and delivered Standard Dental Units (SDU-P and SDU-D) as predictors of NAFLD. AUC: Area Under the Curve SDU-P: blue line, SDU-D: green line, reference line-orange.

Receiver operating characteristic (ROC) analyses were performed on the SDU-P and SDU-D as predictors of NAFLD. ROC curves were plotted, and the area under the curve (AUC) was calculated. Figure 1 presents ROC analyses and AUC calculations for NAFLD with SDU-P and SDU-D. As can be seen in Figure 1, the SDU-P exhibited excellent discrimination (0.8 ≥ AUC > 0.7) for NAFLD (AUC = 0.718 (0.703–0.734)), while the SDU-D exhibited poor discrimination (0.6 ≥ AUC > 0.5) for NAFLD (AUC = 0.567 (0.551–0.584)) (Figure 1). Periodontitis diagnosis had a statistically significant positive association with SDU-P (*p* < 0.001), but not with SDU-D (*p* = 0.392) (non-paired *t*-test, data not in a table).

### 3.7. Multivariate Analysis of NAFLD Diagnosis as a Dependent Variable

**Collinearity statistics.** Before executing the multivariate analysis of NAFLD as a dependent variable, we ran a linear regression analysis to examine the collinearity between the independent variables. As can be seen in Table 6, collinearity was ruled out (VIF < 2.5). 

**Multivariate Analysis.** Following the collinearity analysis, we performed a multivariate binary logistic regression analysis for NAFLD diagnosis as the dependent variable (Table 6). The independent variables were entered into the analysis simultaneously. The following parameters maintained a statistically positive association with NAFLD in the multivariate analysis presented in Table 6 (descending order from highest to lowest OR): male sex (OR = 3.91 (2.29–6.66)), hyperlipidemia (OR = 3.69 (2.75–4.95)), diabetes Type 2 (OR = 3.14 (2.21–4.46)), hypertension (OR = 1.67 (1.30–2.14)), periodontitis (OR = 1.42 (1.06–1.89)), BMI (OR = 1.15 (1.13–1.18)), and age (OR = 1.08 (1.06–1.09)) (Table 6).

## 4. Discussion

The present study research analyzed the associations of NAFLD with dental parameters, while controlling for socio-demographic parameters, health-related habits, and each of the MetS components, consequences, and related conditions among a nationally representative sample of 132,529 young and middle-aged adults. The study demonstrated a statistically significant association between NAFLD and periodontitis, even following the multivariate analysis (Table 6). Moreover, the ROC analysis demonstrated that the SDU-P (planned) exhibited excellent discrimination for NAFLD (Figure 1). Periodontitis diagnosis also had a statistically significant positive association with SDU-P, but not with SDU-D. The SDU score considers the time and complexity of the procedure and represents the procedural burden. It can be concluded that SDU-P, but not SDU-D, is a better predictor of NAFLD and periodontitis. In other words, NAFLD and periodontal morbidity are associated with a higher dental treatment needs burden, rather than with dental treatments performed de facto, which is more dependent on external influencing factors, such as patient compliance, availability of treatment, treatment experience, and specialty of the treating dental professional. Overall, the results confirmed the hypothesis of this research, i.e., that NAFLD is associated with dental morbidity, particularly with periodontitis. The study established a profile of the “patient vulnerable to NAFLD”, which, following multivariate analysis, maintained the well-known risk factors for NAFLD, including older age, male sex, and other MetS components, including diabetes type 2, hypertension, hyperlipidemia, BMI, and periodontitis. This profile is in line with the current new definition of metabolic dysfunction-associated fatty liver disease (MAFLD).

### 4.1. Socio-Demographic Parameters and NAFLD

In line with the literature, our findings demonstrate that older age and male sex retained a statistically significant positive association with NAFLD, even following multivariate analysis. Age is associated with a higher prevalence of NAFLD, and disease progression seems to be faster in older patients, with the male gender appearing as a risk factor [38]. In women, it seems that estrogen may play a protective effect against NAFLD [39]. Previous studies also demonstrated a statistically significant association between lower SES [40] and lower education, with a higher NAFLD prevalence. However, our univariate results should be interpreted with the utmost caution, since SES, locality of residence, and birth countries lost statistical significance with NAFLD following multivariate analysis, and education did not enter the multivariate analysis, due to its collinearity with age.

### 4.2. Health-Related Habits and NAFLD

Previous studies have shown that NAFLD is positively associated with passive and heavy active smoking, and active smoking and BMI have been shown to have a synergistic effect on prevalent NAFLD [41]. Consumption of simple sugars (glucose and fructose) is a factor attributing to both NAFLD development and dental caries. Chronic fructose exposure can lead to inflammation, liver fat accumulation, NAFLD, and metabolic syndrome [42]. Interestingly, none of the health-related habits retained a statistically significant association with NAFLD following multivariate analysis, reflecting the importance of other parameters, such as socio-demographics, MetS components, and periodontitis, which are all associated with lifestyle habits that serve as confounders, mediators, or common cause.

### 4.3. MetS Components, Consequences, and Related Conditions and NAFLD

Following multivariate analysis, NAFLD maintained a statistically significant positive association with diabetes type 2, hypertension, hyperlipidemia, and BMI in line with the current new definition of metabolic dysfunction-associated fatty liver disease (MAFLD). Indeed, an intimate association exists between MAFLD and type 2 diabetes with over 70% of patients with type 2 diabetes having MAFLD; therefore, type 2 diabetes is now considered as one of the three criteria for defining MAFLD, along with overweight and obesity [2]. Excess body weight has a strong pathological link to MAFLD and is a critical determinant of adverse clinical outcomes [2]. Obesity can be classified as metabolically healthy obesity (MHO) and metabolically unhealthy obesity [43], and therefore, the presence of both excess weight and metabolic dysfunction have independent effects on the risk of MAFLD [2], as were considered in the current study. Other metabolic risk abnormalities included in the new MAFLD diagnosis were also considered in the present study, including plasma triglycerides, HDL, prediabetes, fasting glucose, HbA1c, and CRP (Table 3).

### 4.4. Dental Status and NAFLD

The study demonstrated a statistically significant association between NAFLD and periodontitis, independent of socioeconomic variables, health-related habits, and MetS morbidity. Moreover, the SDU-P (planned), which represents the dental procedural burden, exhibited excellent discrimination for NAFLD. To the best of our knowledge, this study is the first to perform ROC analysis to illustrate the diagnostic ability of the dental procedural burden in NAFLD diagnosis.

NAFLD shares some risk factors with periodontitis and dental caries, such as smoking and the consumption of simple sugars (glucose and fructose) [42]. In agreement, a recent cross-sectional study has shown that untreated caries, adults with <20 teeth, and/or people with moderate-severe periodontitis were more likely to have NAFLD [5]. The number of missing teeth has been also previously shown to be positively associated with a higher prevalence rate of NAFLD in males with over 6 teeth missing, but not in females [44,45]. In the present study, the parameter of missing teeth was positively associated with NAFLD in the univariate analysis, but lost its statistical significance in the multivariate analysis. This could reflect the fact that some of the tooth loss could be attributed to periodontitis, which retained a statistically significant positive association with NAFLD.

Another population-based cohort investigation concluded that a history of periodontitis might be a risk factor for NAFLD [46,47]. Individuals with hepatic disease showed a higher prevalence of periodontal disease, worse oral hygiene, and periodontal health status, compared to healthy patients [48]. In concordance, our results also demonstrated more scaling, root planning sessions, and more appointments with periodontists.

Several hypotheses link NAFLD and periodontitis, through periodontal pathogens, inflammatory mediators, and oxidative stress [49]. In a systematic review, a significant positive association was shown between NAFLD and clinical microbial periodontal parameters and between NAFLD and self-reported categories of tooth loss in males, but not females [50]. Although occurring in distant parts of the body, NAFLD and periodontitis pathologies share some etiological factors regarding their onset and portray an unbalanced inflammatory response in a susceptible host. Some of the proposed effects of P. gingivalis on hepatic cells include inhibiting glycogenesis and triggering a cascade that increases the transcription of pro-inflammatory genes, such as TNF-α and IL-1β. Therefore, the progression of both diseases can be aggravated by insulin resistance and the increased systemic pro-inflammatory profile found in diabetic and obese individuals [26,51,52]. The clinical relevance of the results is that clinicians and health authorities should be aware of the high periodontal morbidity in NAFLD patients and refer them to evaluation by dentists. Therapy requires customized treatment plans to address the specific characteristics of each patient and focus on high-risk populations.

## 5. Strength and Limitations

The main strengths of the current research are its large sample size (132,529 individuals), as well as the strict protocol and uniform sociodemographic, medical, and dental codes used. The records-based design is not influenced by patient recall bias, excluding health-related habits. Furthermore, the large sample size enabled us to capture the consequences of MetS, such as TIA and stroke, which are relatively uncommon among young to middle-aged adults. Moreover, routine evaluation of dental pathologies was based on both clinical and radiological examinations. This enhances the diagnosis of hidden occlusal or interproximal caries.

The limitations of the current research include the cross-sectional design, which cannot address causality and can only suggest the associations and correlations between variables. While the research considered and analyzed important factors and covariates, considering the depth of the issues discussed, other parameters were not considered, such as the severity of periodontitis, genetics and epigenetics parameters, childhood and in utero exposures, and history of health-related habits. While multiple ethnicities among a nationwide sample were included in the study, the study population captures the military personnel in Israel, which may limit the generalizability of the results. There is a need for future multicenter international longitudinal prospective studies that will include both genetic and more epidemiological data in other settings and populations to discover the underlying mechanisms underlying the observed associations found in this study.

## 6. Conclusions

The conclusions of this study are:

- The results confirmed the hypothesis of this research, i.e., that NAFLD is associated with dental morbidity, particularly with periodontitis.
- The multivariate analysis established a profile of the “patient vulnerable to NAFLD”, including older age, male sex, and other MetS components, including diabetes type 2, hypertension, hyperlipidemia, BMI, and periodontitis.
- This profile aligns with the current new definition of metabolic dysfunction-associated fatty liver disease (MAFLD).
- A collaborative effort is needed from the dental and general medical authorities by sharing information regarding dental and systemic morbidities.
- The study also highlights the need to adopt a holistic risk management approach that considers both dental and systemic conditions.

## Figures and Tables

**Table 1:** The Associations between NAFLD and Socio-Demographic Parameters.

Parameter	Variable	Without NAFLD (N, %)	NAFLD (N, %)	*p* Value	OR and 95% CI #
NAFLD diagnosis (No/Yes)		131,591 (99.3)	938 (0.7)		
Sex	Men	98,575 (99.1)	891 (0.9)	<0.001 ^	6.29 (4.69–8.47)
	Women	33,016 (99.9)	47 (0.1)		1
Education (Y)	High school	111,908 (99.8)	204 (0.2)	<0.001 *	1
	Technicians	7081 (95.4)	345 (4.6)		26.73 (22.44–31.83)
	Academic	12,427 (97.0)	389 (3.0)		17.17 (14.48–20.36)
Locality of residence	Urban Jewish	112,635 (99.3)	833 (0.7)	<0.001 *	0.26 (0.16–0.43)
	Urban Non-Jewish	17,835 (99.5)	83 (0.5)		0.16 (0.09–0.28)
	Rural	567 (97.3)	16 (2.7)		1
Socio-economic status (SES)	Low	5677 (99.3)	42 (0.7)	0.005 *	1.19 (0.86–1.65)
	Medium	68,093 (99.2)	526 (0.8)		1.25 (1.09–1.43)
	High	56,359 (99.4)	348 (0.6)		1
Birth country	Western Europe	10,463 (99.0)	108 (1.0)	<0.001 *	1.49 (1.22–1.83)
	Eastern Europe	1701 (99.2)	14 (0.8)		1.19 (0.70–2.02)
	Asia	496 (97.4)	13 (2.6)		3.79 (2.18–6.61)
	Ethiopia	2180 (99.8)	5 (0.2)		0.33 (0.13–0.80)
	Africa	334 (96.8)	11 (3.2)		4.77 (2.60–8.73)
	North America	2854 (99.8)	5 (0.2)		0.25 (0.10–0.61)
	South America	952 (99.5)	5 (0.5)		0.76 (0.31–1.83)
	Israel	112,582 (99.3)	777 (0.7)		1
**Parameter**	**NAFLD**	**Mean ± SD**		*p* value **	**OR (95% CI) ##**
Age	No	21.78 ± 5.88		<0.001	1.19 (1.18–1.20)
	Yes	36.81 ± 7.67			
Time in service	No	3.02 ± 6.13		<0.001	1.14 (1.13–1.15)
	Yes	18.46 ± 9.17			

^ Pearson Chi-Square; * likelihood ratio, ** non-paired *t*-test, # binary logistic regression; ## generalized linear models.

**Table 2:** The Associations between NAFLD and Metabolic Syndrome (MetS) related conditions.

Parameter	Variable	No NAFLD	NAFLD	*p* Value ^	OR and 95% CI #
Hypertension	No	128,473 (99.5)	693 (0.5)	<0.001	1
	Yes	3118 (92.7)	245 (7.3)		13.51 (11.76–15.62)
Hyperlipidemia	No	124,527 (99.8)	276 (0.2)	<0.001	1
	Yes	7064 (91.4)	662 (8.6)		38.46 (33.33–45.45)
Impaired glucosetolerance (IGT)	No	131,488 (99.3)	913 (0.7)	<0.001	1
	Yes	103 (80.5)	25 (19.5)		34.95 (22.47–54.37)
Diabetes type 2	No	131,321 (99.3)	863 (0.7)	<0.001	1
	Yes	270 (78.3)	75 (21.7)		33.33 (27.02–41.66)
Obesity	No	124,826 (99.8)	255 (0.2)	<0.001	1
	Yes	6765 (90.8)	683 (9.2)		45.45 (38.46–52.63)
Cardiovascular disease	No	128,161 (99.4)	770 (0.6)	<0.001	1
	Yes	3430 (95.3)	168 (4.7)		7.81 (6.62–9.17)
Obstructive sleep apnea (OSA)	No	131,308 (99.3)	903 (0.7)	<0.001	1
	Yes	283 (89.0)	35 (11.0)		16.13 (11.76–22.22)
Stroke	No	131,509 (99.3)	928 (0.7)	<0.001	1
	Yes	82 (89.1)	10 (10.9)		15.63 (8.62–27.78)
Transient ischemic attack (TIA)	No	131,500 (99.3)	930 (0.7)	<0.001	1
	Yes	91 (91.9)	8 (8.1)		11.49 (5.92–22.22)

^ Pearson Chi-Square; # binary logistic regression.

**Table 3:** The Associations of NAFLD with Auxiliary Test results.

Parameter	Without NAFLD		NAFLD		*p* Value *	OR and 95% CI ##
	N	Mean ± SD	N	Mean ± SD		
Weight (kilograms)	65,810	73.04 ± 32.44	807	93.95 ± 17.22	<0.001	1.005 (1.004–1.005)
Body mass index (BMI)	65,589	24.20 ± 4.24	805	30.28 ± 4.64	<0.001	1.231 (1.217–1.245)
C reactive protein (CRP) (mg/L)	29,878	3.74 ± 10.12	540	5.54 ± 12.83	<0.001	1.010 (1.005–1.015)
Glycated hemoglobin (HbA1c) (%)	1727	5.35 ± 0.92	216	5.78 ± 1.18	<0.001	1.277 (1.143–1.427)
Fasting glucose (mg/dL)	2421	86.95 ± 11.65	106	91.00 ± 16.79	0.016	1.020 (1.008–1.032)
Cholesterol (mg/dL)	27,280	175.43 ± 35.48	900	187.79 ± 37.96	<0.001	1.009 (1.007–1.011)
High-density lipoprotein (HDL) (mg/dL)	27,273	48.49 ± 11.79	900	41.81 ± 8.83	<0.001	0.938 (0.931–0.945)
Low-density lipoprotein (LDL) (mg/dL)	19,359	108.05 ± 29.92	854	114.95 ± 31.85	<0.001	1.007 (1.005–1.009)
LDL cholesterol calculated (mg/dL)	16,788	108.10 ± 30.26	670	115.18 ± 33.10	<0.001	1.007 (1.005–1.009)
Triglycerides (mg/dL)	27,283	102.75 ± 62.00	900	156.49 ± 95.03	<0.001	1.007 (1.006–1.008)
Very low-density lipoprotein (VLDL) (mg/dL)	27,234	20.30 ± 10.93	897	29.90 ± 14.67	<0.001	1.051 (1.047–1.055)
Non-HDL cholesterol (mg/dL)	16,035	128.76 ± 34.82	787	144.69 ± 35.39	<0.001	1.012 (1.010–1.014)

* non-paired *t*-test, ## generalized linear models.

**Table 4:** The Associations of NAFLD with Health-Related Habits and Attendance Patterns.

Parameter	Variable	Without NAFLD	NAFLD	*p* Value ^	OR and 95% CI #
Smoking	No	125,060 (99.5)	585 (0.5)	<0.001	1
	Yes	6531 (94.9)	353 (5.1)		11.55 (10.10–13.2)
Teeth brushing once a day or more	No	17,557 (98.5)	263 (1.5)	<0.001	1
	Yes	39,381 (99.3)	295 (0.7)		0.50 (0.42–0.59)
Consumption of a cariogenic diet	No	34,106 (98.8)	415 (1.2)	<0.001	1
	Yes	22,832 (99.4)	143 (0.6)		0.52 (0.43–0.62)
Consumption of sweetened beverages	No	32,601 (98.8)	408 (1.2)	<0.001	1
	Yes	24,337 (99.4)	150 (0.6)		0.49 (0.41–0.59)
**Parameter**	**Variable**	**Without NAFLD**	**NAFLD**	*p* Value **	**OR and 95% CI ##**
Total number of appointments with a general physician		14.19 ± 11.96	17.85 ± 15.01	<0.001	1.016 (1.016–1.024)
The number of times where scaling was performed		0.63 ± 0.86	1.07 ± 1.22	<0.001	1.41 (1.35–1.47)
The number of times where root planning was performed		0.06 ± 0.57	0.47 ± 1.56	<0.001	1.35 (1.30–1.40)
The number of examinations by an oral medicine specialist		0.01 ± 0.17	0.07 ± 0.43	<0.001	1.59 (1.38–1.82)
The number of examinations by a periodontal specialist		0.03 ± 0.27	0.21 ± 0.68	<0.001	1.84 (1.70–1.99)
Total number of dental appointments		5.81 ± 10.14	15.26 ± 18.16	<0.001	1.03 (1.02–1.03)
Non-attendance to scheduled dental appointments		0.98 ± 2.646	2.17 ± 4.218	<0.001	1.06 (1.05–1.08)

^ Pearson Chi-Square; ** non-paired *t*-test, # binary logistic regression; ## generalized linear models.

**Table 5:** The Associations between NAFLD and Dental Status.z.

Parameter	Variable	Without NAFLD	NAFLD	*p* Value ^	OR and 95% CI #
Periodontitis	No	51,424 (99.1)	442 (0.9)	<0.001	1
	Yes	5514 (97.9)	116 (2.1)		2.41 (1.97–2.96)
Presence of an oral soft tissue lesion in an oral examination	No	130,013 (99.3)	859 (0.7)	<0.001	1
	Yes	1578 (95.2)	79 (4.8)		7.25 (5.81–9.09)
**Parameter**		**Without NAFLD**	**NAFLD**	*p* Value **	**OR and 95% CI ##**
		**Mean ± SD**	**Mean ± SD**		
The number of teeth that required one surface amalgam filling		0.61 ± 1.21	0.39 ± 0.88	<0.001	0.82 (0.76–0.88)
The number of teeth where one surface amalgam filling was performed		0.29 ± 0.78	0.26 ± 0.70	<0.009	0.94 (0.87–1.03)
The number of teeth that required two amalgam fillings on two surfaces		2.03 ± 1.46	1.67 ± 1.12	<0.001	0.79 (0.71–0.88)
The number of teeth where two surface amalgam fillings were performed		0.32 ± 0.84	0.47 ± 0.86	<0.001	1.17 (1.10–1.24)
The number of teeth that required three and more amalgam fillings on surfaces		0.11 ± 0.45	0.14 ± 0.47	<0.035	1.14 (1.01–1.28)
The number of teeth where three and more surface amalgam fillings were performed		0.07 ± 0.32	0.13 ± 0.40	<0.001	1.44 (1.27–1.64)
The number of teeth that required four or more surfaces amalgam fillings		0.01 ± 0.11	0.02 ± 0.15	<0.025	1.72 (1.22–2.43)
The number of teeth where four or more surfaces amalgam fillings were performed		0.03 ± 0.18	0.07 ± 0.31	<0.001	1.91 (1.58–2.31)
The number of teeth that required resin-based composite fillings on one to four surfaces, anterior		0.24 ± 0.79	0.30 ± 0.93	<0.057	1.08 (1.01–1.15)
The number of teeth where resin-based composite fillings were performed on one to four surfaces, anterior		0.25 ± 0.86	0.45 ± 2.14	<0.005	1.11 (1.07–1.15)
Total number of teeth that required fillings		1.55 ± 2.41	1.42 ± 2.13	<0.048	0.97 (0.94–1.00)
Total number of teeth where fillings were performed		1.00 ± 1.96	1.49 ± 2.81	<0.001	1.08 (1.06–1.10)
Total number of teeth that required endodontic treatment		0.08 ± 0.38	0.13 ± 0.41	0.001	1.21 (1.09–1.34)
Total number of teeth where endodontic treatment was performed		0.07 ± 0.33	0.18 ± 0.48	<0.001	1.53 (1.39–1.69)
The number of teeth that required prefabricated (direct, post and core)		0.10 ± 0.42	0.15 ± 0.43	0.002	1.20 (1.08–1.34)
The number of teeth on which prefabricated (direct, post and core) was performed		0.10 ± 0.39	0.25 ± 0.61	<0.001	1.44 (1.341. 55)
The number of teeth that required crowns		0.15 ± 0.57	0.26 ± 0.70	<0.001	1.22 (1.14–1.30)
The number of teeth where a crown was performed		0.05 ± 0.53	0.34 ± 1.40	<0.001	1.21 (1.16–1.25)
The number of teeth that required extractions		0.14 ± 0.51	0.22 ± 0.71	0.001	1.23 (1.13–1.34)
Total number of teeth where extractions were performed		0.10 ± 0.42	0.23 ± 0.79	<0.001	1.27 (1.19–1.36)
Missing teeth		0.57 ± 1.28	1.30 ± 1.67	<0.001	1.15 (1.12–1.18)

^ Pearson Chi-Square; ** non-paired *t*-test, # binary logistic regression; ## generalized linear models.

**Table 6:** **Multivariate Logistic Regression Analysis** and **Collinearity Statistics** for NAFLD diagnosis as the dependent variable with statistically significant independent parameters.

Parameter		Multivariate Logistic Regression Analysis				Linear Regression Analysis Collinearity Statistics	
		B	Std. Error	*p* Value	OR (95% CI)		
						Tolerance	VIF
(Intercept)		**7.19**	**0.98**	**<0.001**	**0.001 (0.000–0.005)**		
Age		0.07	0.008	**<0.001**	1.08 (1.06–1.09)	0.448	2.234
Sex: men vs. women		1.36	0.27	**<0.001**	3.91 (2.29–6.66)	0.934	1.071
Locality of residence (reference: rural)	urban Jewish	−0.24	0.56	0.667	0.78 (0.26–2.35)	0.998	1.012
	Urban non-Jewish	−0.69	0.58	0.233	0.49 (0.16–1.56)	0.979	1.021
Socioeconomic status (SES) (reference: low SES)	High	0.12	0.25	0.619	1.13 (0.68–1.87)	0.948	1.055
	Medium	0.32	0.24	0.183	1.39 (0.85–2.25)	0.944	1.059
Birth country (reference native Israelis)	Western Europe	0.19	0.16	0.254	1.21 (0.87–1.671	0.983	1.017
	Eastern Europe	−0.11	0.60	0.856	0.89 (0.27–2.93)	0.979	1.022
	Asia	−0.45	0.56	0.417	0.63 (0.21–1.90)	0.995	1.005
	Ethiopia	0.07	0.60	0.897	1.08 (0.33–3.54)	0.984	1.017
	Africa	0.42	0.48	0.382	1.52 (0.59–3.94)	0.995	1.005
	North America	0.08	0.60	0.883	1.09 (0.33–3.55)	0.987	1.013
	South America	0.02	0.61	0.969	1.02 (0.31–3.39)	0.997	1.003
Smoking		0.15	0.11	0.183	1.16 (0.93–1.45)	0.749	1.334
Teeth brushing once a day or more		0.05	0.13	0.696	1.05 (0.81–1.36)	0.694	1.441
Consumption of a cariogenic diet		0.03	0.16	0.830	1.03 (0.75–1.43)	0.587	1.702
Sweetened beverages		−0.23	0.16	0.145	0.79 (0.57–1.08)	0.576	1.736
Diabetes type 2		1.14	0.18	**<0.001**	3.14 (2.21–4.46)	0.917	1.090
Impaired glucose tolerance (IGT)		0.51	0.42	0.224	1.67 (0.73–3.85)	0.963	1.038
Hypertension		0.51	0.12	**<0.001**	1.67 (1.30–2.14)	0.901	1.109
Hyperlipidemia		1.31	0.15	**<0.001**	3.69 (2.75–4.95)	0.546	1.832
BMI		0.14	0.01	**<0.001**	1.15 (1.13–1.18)	0.840	1.190
Cardiovascular disease		0.02	0.14	0.901	1.02 (0.76–1.36)	0.920	1.086
Obstructive sleep apnea (OSA)		−0.39	0.30	0.185	0.67 (0.37–1.21)	0.972	1.029
Periodontitis		0.35	0.14	**0.017**	1.42 (1.06–1.89)	0.959	1.043
Missing teeth		−0.02	0.03	0.513	0.97 (0.91–1.04)	0.768	1.302
Total number of teeth that required endodontic treatment		−0.04	0.11	0.740	0.96 (0.76–1.21)	0.810	1.235
The number of teeth that required regular extraction		−0.11	0.07	0.149	0.89 (0.77–1.04)	0.949	1.053
The number of teeth that required a crown		−0.006	0.05	0.924	0.99 (0.88–1.11)	0.822	1.216
Presence of oral soft tissue disease		0.03	0.19	0.873	1.03 (0.71–1.50)	0.854	1.123

Statistically significant values are in bold. VIF: variance inflation factor.

## Data Availability

Data sharing not applicable.

## Abbreviations

BMI	Body mass index
MPR	Medical patient record
CRP	C-reactive protein
CVD	Cardiovascular disease
DPR	Dental patient record
HbA1c	Glycated hemoglobin
HDL	High-density lipoprotein
IDF	Israeli Defense Force
LDL	Low-density lipoprotein
MetS NAFLD	Metabolic syndrome Non-alcoholic fatty liver disease
OSA	Obstructive sleep apnea
SES	Socio-economic status
TIA	Transient ischemic attack
VLDL	Very low-density lipoprotein
